# Efficacy of a Cognitive Behavioral Intervention for the Prevention of Depression in Nonprofessional Caregivers Administered through a Smartphone App: A Randomized Controlled Trial

**DOI:** 10.3390/jcm12185872

**Published:** 2023-09-09

**Authors:** Fernando L. Vázquez, Vanessa Blanco, Isabel Hita, Ángela J. Torres, Patricia Otero, Mario Páramo, Mar Salmerón

**Affiliations:** 1 Department of Clinical Psychology and Psychobiology, Faculty of Psychology, Rúa Xosé María Suárez Núñez, s/n, Campus Vida, University of Santiago de Compostela, 15782 Santiago de Compostela, Spain; 2 Department of Evolutionary and Educational Psychology, Faculty of Psychology, Rúa Xosé María Suárez Núñez, s/n, Campus Vida, University of Santiago de Compostela, 15782 Santiago de Compostela, Spain; 3 Department of Psychiatry, Radiology, Public Health, Nursing and Medicine, Faculty of Medicine, Rúa de San Francisco, s/n, University of Santiago de Compostela, 15782 Santiago de Compostela, Spain; 4 Department of Psychology, Faculty of Educational Studies, Campus de Elviña, s/n, University of A Coruña, 15071 A Coruña, Spain; 5 Psychiatry Service, University Hospital Complex of Santiago de Compostela, R/ Ramón Baltar, s/n, Galician Health Service [SERGAS], 15706 Santiago de Compostela, Spain

**Keywords:** depression, prevention, app, smartphone, caregiver, cognitive behavioral, randomized controlled trial

## Abstract

Due to the limited availability of in-person interventions for caregivers, the development of effective programs that use new technologies to prevent depression is needed. The goal of this research was to assess the efficacy of a cognitive behavioral intervention for the prevention of depression, administered to nonprofessional caregivers through a smartphone application (app). One hundred and seventy-five caregivers were randomly assigned to either an app-based cognitive behavioral intervention (CBIA), the CBIA intervention plus a telephone conference call (CBIA + CC), or an attention control group (ACG). At post-intervention, the incidence of depression was lower in the CBIA and CBIA + CC compared to the ACG (1.7% and 0.0% vs. 7.9%, respectively). The absolute risk, relative risk, and number needed to treat compared to the ACG were 6.2%, 21.6%, and 16 for the CBIA, whilst they were 8%, 0.0%, and 13 for the CBIA + CC. Depressive symptomatology was significantly lower in the CBIA and CBIA + CC compared to the ACG (*d* = 0.84, Cliff’s *δ* = 0.49; *d* = 1.56, Cliff’s *δ* = 0.72), as well as in the CBIA + CC compared to the CBIA (*d* = 0.72, Cliff’s *δ* = 0.44). The prevention of depression was more likely in participants who received the CBIA, and adding the conference call in the CBIA + CC group improved the likelihood of this.

## 1. Introduction

Increasing life expectancies have led to an aging population and an associated increase in disabilities. Disabilities are conceptualized by the International Classification of Functioning, Disability and Health (ICF [1]) as the result of a complex dynamic interaction between a person’s health condition, which refers to an acute or chronic disease, disorder, trauma, or injury, and his/her contextual factors, which include environmental and personal variables and are a reflection of particular life circumstances. Worldwide, 15% of the population has a disability [2], a figure that rises to 33.3% among those over 50 years old in low- and middle-income countries [3]. Many of these people need daily help, which highlights the importance of nonprofessional caregivers. Nonprofessional caregivers are persons close to a patient (e.g., family members, relatives) who provide unpaid care by performing a large variety of care tasks, such as emotional support and assistance [4]. Of the European and Spanish populations, 34.3% and 29.2%, respectively, are nonprofessional caregivers [5], a demanding role that is associated with physical and mental health issues [6,7]. Depression is a particularly notable outcome; in this population, previous findings indicate that depressive disorders are a result of the combination of stressful life events with vulnerability and psychological factors [8]. According to the results of longitudinal studies, depressive symptoms are the consequence of appraising the caregiving situation as highly stressful [9], and there is a strong relationship between the restriction of social and recreational activities and a higher severity of depressive symptoms [10]. Researchers have found that the aggregate prevalence of depression in caregivers ranges between 34.0% [7] and 42.3% [11], with 40.2% presenting depressive symptoms [12]. These figures are alarming due to the deleterious effects that depression can have on quality of life, productivity, and the fulfillment of important roles [13]. Furthermore, depression interferes with the quality of care [14].

Therefore, the prevention of depression in this group is essential. One particularly promising approach is indicated prevention, aimed at people who present signs and symptoms of major depression but do not meet the full diagnostic criteria [15]. The limited number of randomized controlled trials (RCTs) that have examined the efficacy of indicated prevention programs for depression in caregivers have found encouraging results for programs administered in-person [16,17,18] and via conference calls [19]. However, these formats have barriers. In-person programs reach fewer users and present challenges related to transportation, balancing commitments, and time, while conference call formats can mean long phone calls and scheduling difficulties. The use of smartphone apps is one way to address these obstacles: they are readily accessible, given that 83% of the world population had a mobile phone with broadband in 2021 [20], and they allow participants to receive the service and review materials on their own schedules. 

Despite this, only one RCT [21] has examined the efficacy of an app used to prevent depression in Australian workers, and found a lower incidence of depression and post-intervention depressive symptomatology. Only one RCT [22] has evaluated the efficacy of an app for stress among caregivers, showing a reduction in depressive symptoms. However, to our knowledge there is no RCT that has evaluated an app-based intervention for the indicated prevention of depression, or for the prevention of depression in caregivers.

One limitation of mental health apps has been poor adherence (e.g., [23]), which can be remedied by including human support [24]. Adding regular phone contact can increase adherence and reduce dropouts [25,26,27,28]; however, the type of support that is the most efficacious has yet to be examined. Positive and corrective feedback is a versatile psychological tool for imparting cognitive behavioral skills, which consists of reinforcing the correct performance of a certain behavior and providing instructions to change those behaviors that have been performed incorrectly, and could improve adherence as well as efficacy [29] by allowing the strengthening of the therapeutic alliance, providing support and encouragement, and monitoring the assignment of homework [30].

The goal of this research was to assess the efficacy of an app-based cognitive behavioral intervention for the indicated prevention of depression in nonprofessional caregivers with and without contact via a conference call. We expected caregivers receiving both interventions to demonstrate significant post-intervention differences in the incidence of depression and depressive symptomatology compared to an attention control group. The secondary objective was to analyze the role of adherence as a moderator of the effects of the interventions. We expected that the number of modules completed and the percentage of completed intersession tasks would be moderators of the effect of the interventions.

## 2. Materials and Methods

Below we highlight the most important aspects of the methodology, which is described in greater detail in Vázquez et al. [31].

### 2.1. Design

**Figure 1:**
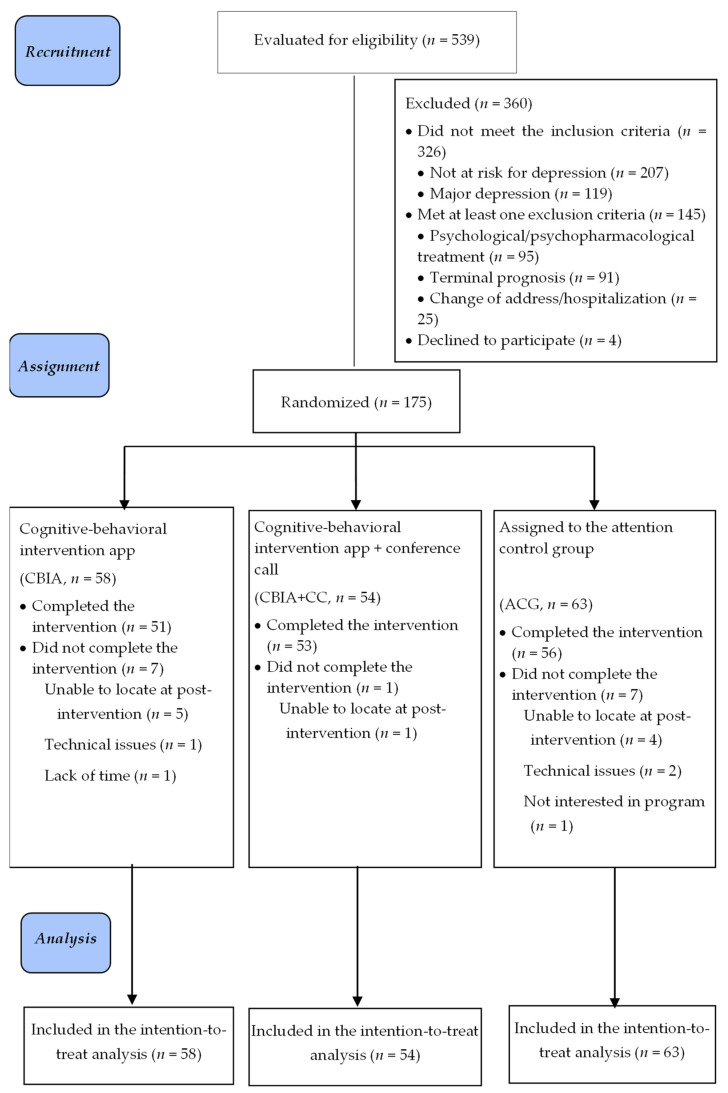
CONSORT 2010 flow diagram.

A three-arm RCT [31] was conducted (Figure 1): (a) a cognitive behavioral intervention using a smartphone app (CBIA); (b) a cognitive behavioral intervention using a smartphone app + conference call with supportive contact (CBIA + CC); and (c) an attention control group (ACG). A statistician outside the research group randomly assigned participants on a 1:1:1 basis to the conditions via the use of a table of computer-generated random numbers. The randomization sequence was communicated to the investigators in closed numbered envelopes—one per participant—with guidelines to use them in numerical sequence.

The researchers evaluated the variables established in the study protocol before and after the intervention. Trained interviewers who were unaware of the study objectives, administered interventions, and group randomization completed the heteroadministered interview over the phone; the participants of the three groups completed the self-administered measures through the app.

### 2.2. Participants

The participants were recruited from public institutions and chronic disease associations in Galicia, a region in northwestern Spain with 2,701,819 residents. The inclusion criteria were as follows: (a) being a nonprofessional caregiver of a dependent person; (b) experiencing high depressive symptomatology (score of ≥16 on the Spanish version of the Center for Epidemiological Studies Depression Scale (CES-D) [32]); (c) not meeting the DSM-5 [33] criteria for a current or past major depressive episode; (d) having a smartphone; and (e) agreeing to take part in all assessments.

The exclusion criteria were as follows: (a) received psychological or psychopharmacological treatment in the last two months; (b) having other disorders that could act as confounding factors (i.e., symptoms due to substance use or medical conditions); (c) having serious mental or medical disorders that required immediate intervention or made it impossible to participate; (d) an imminent terminal prognosis of the care recipient; and (e) anticipating a change of address/institutionalization of the care recipient during the study.

The research was conducted in accordance with the Declaration of Helsinki and was approved by the corresponding Bioethics Committee. Participation was voluntary, and no incentives were offered. Confidentiality was guaranteed, and all of the participants gave informed consent.

### 2.3. Sample Size

On the basis of previous work [18], this research was designed to detect a difference of 18.6% in the cumulative incidence of depressive episodes between the experimental and control groups. The sample size calculation was conducted with GPower (version 3.1.9.7. [34]). Based on studies evaluating apps to treat depression [35], we assumed an alpha of 0.05, a power of 80%, and a sample loss of 15%; therefore, the estimated sample size needed was 174 caregivers (approximately 58 per group). As Figure 1 shows, 539 caregivers were evaluated; 360 (66.8%) did not meet the eligibility criteria and 4 (2.2%) refused to participate. The final sample consisted of 175 caregivers, assigned to three groups: 58 to the CBIA, 54 to the CBIA + CC, and 63 to the ACG. Fifteen participants (8.6%) dropped out, seven in the CBIA, one in the CBIA + CC, and seven in the ACG, for the following reasons: could not be contacted post-intervention, technological problems, lack of time, and lack of interest.

### 2.4. Instruments 

We used the Caregiver and Care Situation Characteristics Questionnaire developed for previous works (e.g., [18]) to assess the sociodemographic (sex, age, marital status, social class, monthly family income, educational level, main activity, and area of residence), care situation, and care recipient variables (number of care recipients, sex and age, relationship, illness, duration of care, daily hours of care, and degree of dependence). We used the Structured Clinical Interview for DSM-5 Disorders, Clinical Version (SCID-5-CV [36]) to assess major depressive episodes (current or past), with a kappa of 0.87 and 0.66 for current and past episodes, respectively [37]. The Spanish version of the CES-D [32] was used to evaluate depressive symptoms, with a Cronbach’s alpha of 0.89. We recorded dropouts, the number of modules completed, and intersession tasks performed through the app to evaluate acceptability. The Spanish version of the Client Satisfaction Questionnaire (CSQ-8; [38]) was used to assess satisfaction, with a Cronbach’s alpha of 0.80. The SCID-V-CV was heteroadministered, while the rest of instruments were self-administered.

### 2.5. Intervention and Control Groups

Prior to the study a protocol was developed, and the interventions were manualized. Subsequently, a pilot study was conducted to evaluate its feasibility and acceptability [39]. 

The CBIA was adapted from an indicated depression prevention intervention based on the model of Lewinsohn et al. [40], which had demonstrated short- and long-term efficacy in in-person and conference call formats [17,18,19]. It was adapted to administration through an app with five 60 min (±20) modules to be completed in five weeks, which taught participants behavioral and cognitive skills that included mood monitoring, relaxation, self-reinforcement, planning pleasant activities, behavior contracts, techniques to increase positive thoughts as well as reduce negative ones, and assertiveness (Table 1).

Participants in the CBIA + CC also received contact via weekly 30 min conference call sessions for each module (in groups of 5–6 caregivers). Positive and corrective feedback [29] was used in the conference call sessions. The positive feedback consisted of providing information on the correct performance of intersession tasks and reinforcement, and the corrective feedback identified tasks that were not performed properly, suggesting changes to improve performance. The CC component was administered by three psychologists (with master’s or doctoral degrees) trained by two mental health professionals with more than 25 years of experience in cognitive behavioral therapy. This training consisted of 35 h of theoretical and practical seminars and role playing. The protocol adherence was 93%.

The ACG also received five modules through an app that focused only on information on informal caregiving, depression, and how to maintain mental health.

### 2.6. Data Analysis

Analyses were performed using SPSS (version 27.0 [41]) and R [42], following the principle of intention to treat (Consolidated Standards of Reporting Trials (CONSORT); [43]). Missing values were imputed using the multiple imputation method with the EMB algorithm in the Amelia II program [44], resulting in 15 imputations. An imputation model was determined for each variable (which did not exceed 10% of intervals that exclude the straight line *y* = *x*) by over-imputation. The mice and miceadds packages were used to combine the model and test parameters [45,46].

We analyzed the cumulative incidence of depression, absolute relative risk reduction (ARR), relative risk (RR), and number needed to treat (NNT) for each group at post-intervention via the use the formulae of Guyatt et al. [47]. The confidence interval (CI) for ARR was estimated according to Wilson’s method [48,49].

We used regression models to compare the depressive symptomatology between groups as well as between pre- and post-intervention. Specifically, linear mixed models (LMMs [50,51]) were used. The D2 statistic was calculated via the use of the results of the analysis of variance (ANOVA) tables of the 15 imputed databases [52]. The Bonferroni correction and Holm–Bonferroni method were used in the a posteriori contrasts (between times, between groups, and interaction between time and group) to correct for multiple comparisons. The effect size was calculated using Cohen’s *d* [53], considering the effect sizes of *d* = 0.2 to be small, *d* = 0.5 to be moderate, and *d* ≥ 0.8 to be large, and Cliff’s delta [54,55], a measure of the effect size for the difference in medians, which is more adequate when the data distribution deviates from normal.

To analyze the potential effect of the variables for which there were significant differences between arms at the baseline (i.e., caregiver age, years caring), the LMM [50,51] was replicated, including those characteristics that were adjustment variables.

Lastly, to obtain the noninferiority limits of the CBIA vs. CBIA + CC, Vázquez et al. [19] was used as a reference, considering CBCC as the active control. The maximum noninferiority margin was conceptualized as the minimum value of the 95% CIs for the measure of effect. For depressive episodes, the ARR between the control and active control was determined: 7.279% (95% CI 0.4583%, 14.10%). For depressive symptoms, the mean difference between the control and active control at post-intervention was 9.2 (95% CI 6.838, 11.562). The noninferiority margin was weighted by a factor, f (0.5), obtaining a weighted operating margin of 0.229 for the ARR and a figure of 3.42 for the difference in means at post-intervention. 

To compare the acceptability and satisfaction of the interventions, Student’s *t*-tests for independent samples were used for continuous variables, while chi-square or Fisher–Freeman–Halton exact tests were used for categorical variables.

In order to analyze the role of adherence (number of modules completed and percentage of completed intersession tasks) as a moderator, moderation analyses were conducted on the 15 databases obtained after imputation analysis, combining the results of the models following Rubin’s rules [56]. The potential moderators were centered following the guidelines recommended by Kraemer and Blasey [57]. The approach selected to evaluate the potential moderating effect is based on the linear regression model proposed by Baron and Kenny [58], *Y* = *α* + *β*1*X* + *β*2*M* + *β*3*XM*, where *Y* represents post-intervention depressive symptoms; *X* represents the group; *M* represents the potential moderator; *β*1 and *β*2 represent the main effects of the group and moderator, respectively; and *β*3 represents the main effect of the interaction. If the *β*3 coefficient was significant, it would indicate the moderating effect of the variable on the differences in depressive symptoms depending on the group.

## 3. Results

### 3.1. Characteristics of the Participants

Table 2 presents the sociodemographic, care situation, and care recipient characteristics: 92.6% were women, with a mean age of 50.0 years (*SD* = 9.8); 68.0% were married/lived with a partner; 49.2% belonged to the middle class; 53.2% had a monthly income of EUR 1000–1999; 41.1% had a primary school education; 52.6% were self-employed or employees; 56.0% were from urban areas; and 72.6% cared for one care recipient. Of the care recipients, 56.6% were women, with a mean age of 52.7 years (*SD* = 31.7); 38.3% were the mother or father of the caregiver; and 50.2% had a mental disorder, neurological disease, or brain damage. Caregivers had provided care for 13.1 years (*SD* = 9.8) and 16.4 h a day (*SD* = 7.4). In terms of the degree of dependency, 60.0% of the recipients scored <50. There were significant differences between conditions for caregiver age, *F* (2, 172) = 3.973, *p* = 0.021, and years caring, *F* (2, 172) = 3.475, *p* = 0.033, but not for other characteristics. 

### 3.2. Incidence of Depression

At post-intervention, 1 of the 58 CBIA caregivers (1.7%), none of the CBIA + CC caregivers, and 5 of the 63 ACG caregivers (7.9%) had developed a depressive episode. The absolute risk reduction (ARR) of the CBIA vs. the ACG was 6.2%, the relative risk (RR) was 21.6%, and the number needed to treat (NNT) was 16. The ARR of the CBIA + CC vs. the ACG was 8%, the RR was 0%, and the NNT was 13. The ARR of the CBIA + CC vs. the CBIA was 1.7%, the RR was 0%, and the NNT was 58. No participant characteristics had significant effects on the incidence of depression. The noninferiority between the CBIA and the CBIA + CC was an ARR of 1.7 (95% CI −5.07, 9.14). Since the 95% CI includes 0 and its upper limit crosses the noninferiority margin, we could not conclude the CBIA’s noninferiority.

### 3.3. Depressive Symptoms

**Figure 2:**
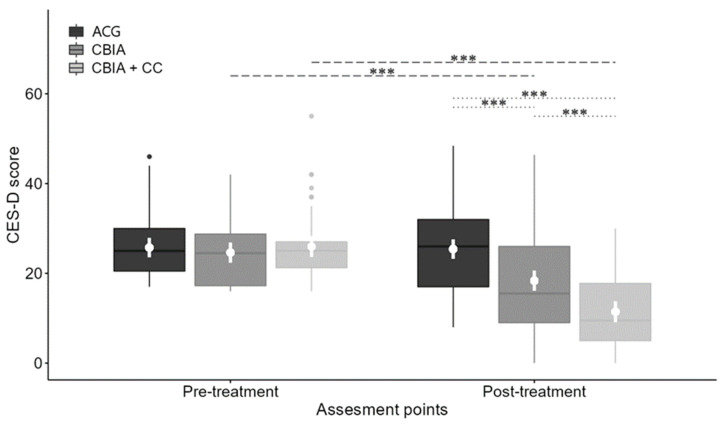
Evolution of depressive symptoms in the three groups. Note: Box plot showing the evolution of depressive symptoms in the three groups. The mean and 95% confidence interval for the CES-D score are shown in a white color inside a box. The long dashed and dotted lines represent, respectively, significant differences within or between groups (***: *p*-value < 0.001).

Figure 2 shows the changes in depressive symptoms in the three groups. The pre- and post-intervention means were, respectively, 24.6 (*SD* = 7.3) and 18.3 (*SD* = 11.3) for the CBIA, 26.0 (*SD* = 11.4) and 11.5 (*SD* = 8.5) for the CBIA + CC, and 25.7 (*SD* = 7.3) and 25.3 (*SD* = 9.8) for the ACG.

Significant effects were found for group (D2 statistic: *F* (2, 1266.120) = 15.981, *p* < 0.001) and time (D2 statistic: *F* (1, 327.686) = 60.061, *p* < 0.001). The CBIA and the CBIA + CC had statistically significant improvements at the post-intervention time point (see Table 3).

The group–time interaction was also significant (D2 statistic: *F* (2, 2058.875) = 16.940, *p* < 0.001). Significant differences were found between the CBIA, CBIA + CC, and ACG, as well as between the CBIA and CBIA + CC at post-intervention (see Table 3).

The noninferiority tests revealed that the difference in mean post-intervention depressive symptomatology between the CBIA and CBIA + CC was 6.93 (95% CI 3.18, 10.68). Since the CI did not cross the no-effect value, and its upper limit crossed the inferiority margin (3.42), the CBIA was inferior to the CBIA + CC.

### 3.4. Impact of Caregiver Age and Years Caring on Depressive Symptoms

To assess if caregiver age and years caring modified the observed effects, they were included in the regression model. The models that included age (*F* (1, 3610) = 0.672, *p* = 0.41) or years caring (*F* (1, 25,034) = 0.788, *p* = 0.37) did not show a significant improvement compared to the models without these variables. In addition, the inclusion of these variables (age and years caring) did not modify the effects observed either in group (*F* (2, 1070) = 16.164, *p* < 0.001; *F* (2, 1417) = 15.798, *p* < 0.001, respectively), time (*F* (1, 325) = 60.029, *p* < 0.001; *F* (1, 321) = 59.937, *p* < 0.001, respectively), or the group–time interaction (*F* (2, 1982) =16.915, *p* < 0.001; *F* (2, 2025) = 16.934, *p* < 0.001, respectively).

### 3.5. Acceptability and Satisfaction

Fifteen caregivers (8.6%) dropped out: seven (12.1%) in the CBIA, one (1.8%) in the CBIA + CC, and seven (11.1%) in the ACG. There were no significant differences between the groups.

The mean number of modules completed was 4.4 (*SD* = 0.8) in the CBIA, 4.7 (*SD* = 0.5) in the CBIA + CC, and 4.0 (*SD* = 1.0) in the ACG; there were significant differences between groups (*U* = 1232, *z* = −2.28, *p* = 0.022). In the CBIA, 31 caregivers (53.4%) completed all of the modules, compared to 39 (72.2%) in the CBIA + CC and 27 (42.8%) in the ACG.

The percentage of completed intersession tasks was 81.7% in the CBIA and 91.1% in the CBIA + CC. The CBIA and CBIA + CC showed a satisfaction of 25.1 (*SD* = 3.3) and 27.9 (*SD* = 3.1), respectively, with significant differences between groups (*U* = 662, *z* = −4.52, *p* < 0.001).

#### Adherence as a Potential Moderator of the Results

The moderation analyses were performed on the depressive symptoms at post-intervention.

The interaction between the number of modules completed and the group is significant for the CBIA vs. the ACG (*β* = −3.79, *p* < 0.001, 95% CI = −5.22–−2.37) and the CBIA + CC vs. the ACG (*β* = −4.12, *p* < 0.001, 95% CI = −6.17–−2.07), showing that it has a moderating effect on depressive symptoms. However, although this moderating effect exists, the differences between groups remain (*t.ratio* = 5.41, *p* < 0.001 for the CBIA vs. the ACG; *t.ratio* = 6.52, *p* < 0.001 for the CBIA + CC vs. the ACG; and *t.ratio* = 2.79, *p* = 0.006 for the CBIA vs. the CBIA + CC).

The interaction between the percentage of tasks and the group is not significant, so we cannot conclude that it has a moderating effect on depressive symptoms.

## 4. Discussion

This study evaluated the efficacy of an app-based cognitive behavioral intervention for the indicated prevention of depression in nonprofessional caregivers, with and without conference call contact. At post-intervention, the appearance of new cases of depression and depressive symptomatology was lower in the intervention groups compared to the control group, with better results in the CBIA + CC. 

The sociodemographic profile of this sample corresponded to a middle-aged married woman who was middle class, with a monthly income of EUR 1000–1999, with a primary school education, who was in the work force, and was from an urban area. The care situation had a typical duration of 13.1 years for 16.4 h a day. The care recipients were predominantly middle-aged women; most of them mothers or fathers of the caregivers; with a mental disorder, neurological disease, or brain damage; and a degree of dependency of <50. This sociodemographic profile partially coincided with that found in previous works that evaluated programs administered in-person [16,17,18], which consisted of middle-aged women with a partner, who were of a low-middle/low social class, with a primary school education, and not in the work force, with a typical care duration of about 10 years and 17 h a day, and in which the care recipients were predominantly women with a mean age close to 80 years old; most of them mothers or fathers of the caregivers; and with a diagnosis of dementia. Likewise, the profile of the participants was partly similar to that found by Vázquez et al. [19] in a randomized controlled trial that assessed interventions administered via conference calls, which corresponded to middle-aged women with a primary school education who were not in the work force, had provided care for 12.8 years and 15.8 h a day, and whose care recipients were mostly their fathers or mothers with dementia. Additionally, it differed from that found by Deady et al. [21], which consisted of men with an average of 40 years old and that were in the work force. Furthermore, the sociodemographic and care characteristics of our sample differed from the profile found by Fuller-Tyszkiewicz et al. [22], which consisted of younger women (average age of 39.5), who did not work outside the home, with a household income of AUS 61,000–100,000, and provided care for more than 40 h a week for a child with a psychosocial disability. 

The incidence of depression was 0.0% in the CBIA + CC and 1.7% in the CBIA, almost five times lower than in the ACG (7.9%), consistent with findings for a conference-call-indicated prevention intervention [19] and another app format intervention [21]. However, these results were lower than those of an in-person-indicated preventive intervention that focused on problem solving [16] and slightly higher than a similar in-person intervention that focused on cognitive behavioral techniques [17]. The findings for the ARR, RR, and NNT indicate that both interventions prevented new episodes of depression but were slightly less efficacious than the results of previous studies on in-person or conference call interventions [16,17,19], but higher than those of Deady et al. [21], with a higher NNT.

In relation to depressive symptomatology, there were differences between groups at post-intervention, with medium/moderate effect sizes between the CBIA and CBIA + CC and large effect sizes for the CBIA + CC and CBIA vs. the ACG. Previous studies [16,17,19] found large effect sizes (similar to the CBIA + CC and CBIA vs. ACG); however, Deady et al. [21] found a small effect size, lower than that for both interventions, and Fuller-Tyszkiewicz et al. [22] found a significant reduction in depressive symptoms in the intervention group from the baseline to post-intervention, which persisted at a 3-month follow-up, but no effect size was reported. The results after controlling for caregiver age and years caring were very similar to those in the unadjusted models, which suggests that these variables did not influence the results.

Taken together, these findings suggest that the effects of the CBIA + CC are like those of other interventions for the indicated prevention of depression in caregivers, and those for the CBIA are slightly smaller than those for the CBIA + CC, but larger than those found in other interventions using apps. The difference between samples could partly explain these findings: the sample in the current study consisted of working women with family responsibilities, which means that they could have benefited more from this app intervention because they had less time to use other resources compared to the samples in the studies by Deady et al. [21] and Fuller-Tyszkiewicz et al. [22]. Additionally, the study sample in Fuller-Tyszkiewicz et al. [22] was composed of mothers who took care of their children with disabilities, which may mean that they have experienced more severe and chronic levels of depressive symptomatology, or be more resistant to treatment, than our sample, composed of daughters that cared for their parents. However, previous research (e.g., [59,60]) has not found differences in depression as a function of the care recipient.

Finally, the dropout rate (8.6%) was higher than that in previous in-person or conference call interventions [16,17,19], but lower than the rate in other studies using apps (i.e., 27% [22] and 52% [21]). The average number of modules completed was similar to that completed in in-person and conference call [17,19] interventions, and higher than that in other app studies [21,22]. Together, the low dropout rate, high number of modules completed, and 80% completion of intersession tasks suggest that the participants found the intervention app acceptable. Previous research has identified the completion of intersession tasks to be as low as one-third of the tasks on average [22]. One explanation for the high acceptability is the use of a simple and accessible intervention that was previously validated in other formats and adjusted to the needs of caregivers. Satisfaction was high, especially for the CBIA + CC, suggesting that supportive contact may have enhanced the quality of the intervention. Though the number of modules completed moderated the effects of the interventions on the depressive symptoms at post-intervention, it did not account for the differences between the three groups, suggesting that the differential effects of the CBIA + CC vs. CBIA could be explained by other variables. A potential candidate to account for these effects could be the group format of the CBIA + CC intervention, which, according to previous research (e.g., [61]), constitutes an important element of therapeutic change.

Though the current study has notable strengths and enhances the current literature—such as being the first RCT to examine the efficacy of an app-based cognitive behavioral intervention for the prevention of depression in caregivers—there are limitations present. Specifically, the lack of follow-ups prevents any measurement of the sustainability of the depressive symptomatology improvements. The mechanisms underlying the effects of the intervention are also unknown. Further research is required to solidify these findings, analyze the sustainability of the effects, examine which specific features of the interventions contribute to greater improvements, and pinpoint the populations that are most likely to benefit from such interventions. This underscores the need for well-designed and rigorous trials to better comprehend the potential of app-based interventions in addressing depression.

The implications of the current study are very salient to research and clinical practice. The app may be an effective tool that is easy to disseminate, which would allow greater service coverage and integration into a staggered care plan for depression in caregivers. Its format enables accessibility and anonymity, reaching a sector of the population that otherwise might not receive help. Adding conference call contact is a simple strategy that increases adherence and efficiency at a reduced cost.

In conclusion, this study supports the efficacy of an app-based cognitive behavioral intervention for the prevention of depression in caregivers, especially when it is complemented by conference call contact using positive and corrective feedback strategies, which improve adherence. In addition, the administration format, simplicity, and low cost mean that it has enormous potential to prevent depression in this population.

## Figures and Tables

**Table 1:** Content of the cognitive behavioral app intervention (CBIA) and the cognitive behavioral app + conference call intervention (CBIA + CC).

Module	Contents
Module 1	Introduction of group members Aim of the program Information on depression and active coping Mood scoring Diaphragmatic breathing training Self-reinforcement Intersession tasks: mood rating, practicing breathing techniques, and self-reinforcement
Module 2	Explanation of the relationship between activities and mood Guidelines and strategies for increasing pleasurable activities Planning of enjoyable activities Behavior contract Intersession tasks: mood rating, practicing breathing techniques, self-reinforcement, and doing planned pleasurable activities
Module 3	Explanation of the relationship between thoughts and mood Thought management techniques (direct approach, priming, and distraction) Planning of enjoyable activities Behavior contract Intersession tasks: mood rating, practicing breathing techniques, self-reinforcement, doing planned pleasurable activities, and practicing thought management techniques
Module 4	Explanation of the relationship between social contact and mood Strategies to increase and improve social relationships Planning enjoyable and social activities Intersession tasks: mood rating, practicing breathing techniques, self-reinforcement, doing planned pleasurable activities, practicing thought management techniques, and making social contact
Module 5	Review of what has been learned. Maintain progress Relapse prevention Good-bye and wrap-up

**Table 2:** Sociodemographic and care characteristics.

Characteristics	Total (*n* = 175)	CBIA (*n* = 58)	CBIA + CC (*n* = 54)	ACG (*n* = 63)
Caregiver Characteristics				
Sex, *n* (%)				
Female	162 (92.6)	52 (89.7)	52 (96.3)	58 (92.1)
Male	13 (7.4)	6 (10.3)	2 (3.7)	5 (7.9)
Average age (*SD*)	50.0 (9.8)	47.6 (9.6)	52.7 (9.5)	49.8 (9.8)
Marital status, *n* (%)				
Single	21 (12.0)	8 (13.8)	4 (7.4)	9 (14.3)
Married/living with a partner	119 (68.0)	37 (63.8)	39 (72.2)	43 (68.3)
Separated/divorced/widowed	35 (20.0)	13 (22.4)	11 (22.4)	11 (17.4)
Social class, *n* (%)				
Low	14 (8.0)	5 (8.6)	3 (5.6)	6 (9.5)
Middle-low	69 (39.4)	24 (41.4)	25 (46.3)	20 (31.7)
Middle	86 (49.2)	27 (46.6)	26 (48.1)	33 (52.4)
Middle-high	6 (3.4)	2 (3.4)	0 (0.0)	4 (6.3)
Net monthly income, *n* (%)				
Up to EUR 999	34 (19.4)	12 (20.7)	12 (22.2)	10 (15.9)
EUR 1000–1999	93 (53.2)	31 (53.4)	29 (53.7)	33 (52.4)
More than EUR 2000	48 (27.4)	15 (25.9)	13 (24.1)	20 (31.7)
Highest academic attainment, *n* (%)				
No formal schooling but can read and write	8 (4.6)	0 (0.0)	3 (5.6)	5 (7.9)
Primary school *	72 (41.1)	28 (48.3)	23 (42.6)	21 (33.4)
Secondary school *	52 (29.7)	17 (29.3)	15 (27.8)	20 (31.7)
University	43 (24.6)	13 (22.4)	13 (24.0)	17 (27.0)
Main activity, *n* (%)				
Self-employed or employee	92 (52.6)	36 (62.1)	28 (51.9)	28 (44.4)
Household chores	68 (38.8)	20 (34.5)	20 (37.0)	28 (44.4)
Retired or unemployed	15 (8.6)	2 (3.4)	6 (11.1)	7 (11.2)
Area of residence, *n* (%)				
Rural	77 (44.0)	30 (51.7)	17 (31.5)	30 (47.6)
Urban	98 (56.0)	28 (48.3)	37 (68.5)	33 (52.4)
Care Situation and Care Recipient Characteristics				
Number of people cared for (*SD*)				
A person	127 (72.6)	44 (75.9)	37 (68.5)	46 (73.0)
More than one	48 (27.4)	14 (24.1)	17 (31.5)	17 (27.0)
Sex of the care recipient, *n* (%)				
Female	99 (56.6)	29 (50.0)	32 (59.3)	38 (60.3)
Male	76 (43.4)	29 (50.0)	22 (40.7)	25 (39.7)
Age of the care recipient (*SD*)	52.7 (31.7)	56.6 (33.3)	49.4 (31.0)	52.0 (30.9)
Relationship of the care recipient to the caregiver, *n* (%)				
Father/mother	67 (38.3)	17 (29.3)	25 (46.3)	25 (39.7)
Spouse/partner	25 (14.3)	9 (15.5)	8 (14.8)	8 (12.7)
Child	57 (32.5)	19 (32.8)	15 (27.8)	23 (36.5)
Other family members	22 (12.6)	11 (19.0)	5 (9.3)	6 (9.5)
Other non-relatives	4 (2.3)	2 (3.4)	1 (1.9)	1 (1.6)
Illness of the person cared for, *n* (%)				
Chromosomal, congenital, and perinatal abnormalities	28 (16.0)	7 (12.1)	10 (18.5)	11 (17.5)
Diseases of the musculoskeletal, connective tissue, cardiovascular, or respiratory systems	26 (14.9)	11 (19.0)	9 (16.7)	6 (9.5)
Mental disorders, neurological diseases, and brain damage	88 (50.2)	28 (48.3)	26 (48.1)	34 (54.0)
Cancer	5 (2.9)	2 (3.4)	3 (5.6)	0 (0.0)
Dementias	28 (16.0)	10 (17.2)	6 (11.1)	12 (19.0)
Average years providing care (*SD*)	13.1 (9.8)	10.4 (8.2)	14.3 (11.0)	14.6 (9.7)
Average daily hours providing care (*SD*)	16.4 (7.4)	16.0 (7.4)	15.2 (7.7)	17.9 (6.9)
Degree of dependence of the care recipient, *n* (%).				
75–100	24 (13.7)	11 (19.0)	8 (14.8)	5 (7.9)
50–74	46 (26.3)	17 (29.3)	13 (24.1)	16 (25.4)
Less than 50	105 (60.0)	30 (51.7)	33 (61.1)	42 (66.7)

Note: CBIA = cognitive behavioral intervention administered through a smartphone app; CBIA + CC = cognitive behavioral intervention administered through a smartphone app plus conference calls; and ACG = attention control group. * Primary school = Spanish General Basic Education (EGB), Basic Technical Training (FPI), or equivalent; Secondary school = general high school, Advanced Technical Training (FPII), or equivalent.

**Table 3:** Student’s t statistics and effect sizes for within-group and between-group effects.

Comparison	t	*p* adj.bonf	*p* adj.holm	Cohen’s *d* (95% CI)	Cliff’s *δ* (95% CI)
Within-Group Effects Tests					
Pre-post CBIA	5.211	<0.001	<0.001	0.87 (0.54, 1.20)	0.50 (0.35, 0.62)
Pre-post CBIA + CC	11.003	<0.001	<0.001	1.76 (1.42, 2.10)	0.77 (0.69, 0.83)
Pre-post ACG	1.076	0.283	0.283	0.17 (−0.14, 0.48)	0.13 (−0.12, 0.32)
Between-Group Effects Tests					
CBIA-ACG	4.207	<0.001	<0.001	0.84 (0.44, 1.23)	0.49 (0.30, 0.63)
CBIA + CC-ACG	7.946	<0.001	<0.001	1.56 (1.16, 1.97)	0.72 (0.61, 0.81)
CBIA-CBIA + CC	3.636	0.001	<0.001	0.72 (0.33, 1.12)	0.44 (0.23, 0.60)

Note: *p*. adj.bonf: *p* adjusted for Bonferroni; *p*. adj.holm: *p* adjusted for Holm–Bonferroni; and CI = confidence interval.

## Data Availability

The data presented in this study are available on request from the corresponding author. The data are not publicly available because they contain sensitive patient information.
